# miR-199b-5p-DDR1-ERK signalling axis suppresses prostate cancer metastasis via inhibiting epithelial-mesenchymal transition

**DOI:** 10.1038/s41416-020-01187-8

**Published:** 2020-11-26

**Authors:** Zhigang Zhao, Shankun Zhao, Lianmin Luo, Qian Xiang, Zhiguo Zhu, Jiamin Wang, Yangzhou Liu, Jintai Luo

**Affiliations:** 1 Department of Urology & Andrology, The First Affiliated Hospital of Guangzhou Medical University; Guangdong Provincial Key Laboratory of Urology, 510230 Guangzhou, China; 2 Department of Urology, Zhejiang Taizhou Central Hospital (Affiliated Hospital of Taizhou University), 318000 Taizhou, China

**Keywords:** Oncogenes, Prostate cancer

## Abstract

**Background:**

The investigation of underlying mechanism and the exploitation of novel therapies for metastatic prostate cancer (PCa) are still urgently needed. miR-199b-5p has been suggested to function as tumour suppressor in various human cancers. However, the clinical significance and role of miR-199b-5p in PCa remain unclear.

**Methods:**

The current study sought to investigate the expression status of miR-199b-5p in PCa and the involved molecular mechanisms in PCa metastasis by using bioinformatics analyses, loss-and gain-of-functions and rescue experiments in vitro and in vivo.

**Results:**

We demonstrated that miR-199b-5p was significantly downregulated in metastatic PCa tissues and cells when compared with the normal prostate tissue, the localised disease, the weakly metastatic and androgen-dependent PCa cell and the normal prostate epithelial cell. We also found that miR-199b-5p drastically suppressed PCa cell proliferation, migration and invasion in vitro and inhibited xenografts tumour growth and metastasis in vivo. Mechanistically, our results showed that miR-199b-5p could inhibit discoidin domain receptor tyrosine kinase 1 (DDR1) expression by directly targeting its 3’-UTR, thereby hindering epithelial-mesenchymal transition (EMT)-associated traits, which were induced by DDR1 activating ERK signalling pathway. Moreover, PCa patients with low miR-199b-5p expression level had a remarkably shorter overall survival than those with high miR-199b-5p level, indicating an association of miR-199b-5p loss with poor prognosis in patients with PCa. Furthermore, DDR1 was upregulated in PCa, and significantly correlated with high Gleason score, advanced pathological stage, tumour metastasis and shorter overall survival.

**Conclusions:**

Our study, for the first time, provide evidence of a tumour-suppressive function of miR-199b-5p in the invasion and metastasis of PCa, supporting the translational exploitation of miR-199b-5p-based therapeutic approaches for PCa metastases. Also, the miR-199b-5p-DDR1-ERK signalling axis identified in this study represents a novel mechanism of regulating EMT in PCa metastases.

## Background

Prostate cancer (PCa) is the most frequently diagnosed malignancy and the second leading cause of cancer-associated death among men in the western countries.^1^ An estimated 1.3 million new cases worldwide have suffered from PCa in 2018.^1^ In China, the incidence and mortality of PCa are getting higher annually.^2^ Although radical prostatectomy or radiotherapy turned out to be effective to control localised PCa, a significant proportion of patients still experience biochemical/local recurrence or metastasis. While advanced PCa is initially controlled with androgen deprivation therapy (ADT), treatment is palliative, and relapse eventually occurs due to the emergence of castration-resistant PCa (CRPC). Moreover, the development of metastasis to distant sites, particularly bone, greatly contributes to high mortality of PCa patients. Unfortunately, no effective therapeutic approaches are currently available for these patients. As such, it is of great importance to better understand the underlying molecular mechanisms contributing to PCa metastasis, which will facilitate the development of the novel therapies or the implementation of currently used approaches in PCa.

MicroRNAs (miRNAs) are endogenous small noncoding RNAs with 17–23 nucleotides that post-transcriptionally repress gene expression through binding to the 3′-untranslated region (3′-UTR) of their target mRNAs.^3^ Accumulating data demonstrated that miRNAs function as novel crucial regulators of diverse biological processes involved in tumorigenesis, invasion and metastasis, and exert the roles of either pro- or antitumour depending on their target genes.^4,5^ Great insights into the implication of miRNAs in the carcinogenesis and metastasis help to better understand the mechanisms of disease and provide the opportunity for a translational exploitation of miRNAs in the development of miRNA-based therapies. Most recently, we found that miR-199b-5p has a significantly prognostic significance in metastasis of patients with PCa.^6^ It has been reported that miR-199b-5p was downregulated in several cancers including hepatocellular carcinoma, renal cell carcinoma, breast cancer and acute myeloid leukaemia, and exerts tumour-suppressive functions in tumour cell growth, invasion and metastasis.^7–10^ A recent study by Zhou et al.^11^ reported that miR-199b-5p overexpression in hepatocellular carcinoma cells induced cell aggregation, reduced cell migration, invasion and TGF-b1-induced epithelial to mesenchymal transition (EMT) in vitro, and suppressed tumour metastasis in vivo. All these findings demonstrated that miR-199b-5p involved in tumour malignant features and progression, suggesting a potential therapeutic target. However, to the best of our knowledge, the clinical significance and functional roles of miR-199b-5p in PCa invasion and metastasis have not been elucidated. In this study, we further investigate the expression status and the underlying mechanisms of miR-199b-5p involved in metastatic PCa and provide evidence for the development of miR-199b-5p-based approaches for PCa.

## Methods

### Patients and tissue samples

In this study, available PCa tissues were collected from 94 consecutive patients with PCa who underwent surgical resection or biopsy at our institution from January 2009 to December 2012, in which 56 patients had primarily localised disease and 38 cases had distal metastases.

### Cell lines and cell culture

The normal human prostate epithelial cell line RWPE-1 and human PCa cell lines LNCaP, DU145, PC-3, VCaP and 22RV1 were obtained from the Type Culture Collection of the Chinese Academy of Sciences (Shanghai, China). Human PCa cell line C4-2 and human embryonic kidney cell 293 T were purchased from the American Type Culture Collection (Manassas, VA, USA). RWPE-1 cells were cultured in the Defined Keratinocyte Serum Free Medium with growth supplement (Gbico, USA). LNCaP, 22RV1, DU145, PC-3, VCaP and 293 T cells were maintained in RPMI-1640 medium (Gbico, USA) or DMEM basic (Invitrogen, USA) supplemented with 10% foetal bovine serum (Gbico, USA) and a 1% penicillin and streptomycin combination. Cell lines were authenticated by short tandem repeat profiling, tested negative for mycoplasma, and used within seven passages between experiments. All cells were grown to 70% confluence prior to experimentation.

### Cell transfection

In this study, for stable transfections, the lentiviral vectors for miR-199b-5p overexpression (named as miR-199b-5p), miR-199b-5p inhibitor (named as anti-miR-199b-5p) and the corresponding miR-negative control (named as NC) were designed and constructed by GenePharma (Shanghai, China), and were transfected into cells at a concentration of 5 × 10^5^/mL seeded in 6-well plates by using Lipofectamine 2000 reagent (Invitrogen, Carlsbad, CA) according to the manufacturer’s protocol. At 48 h post-transfection, the cells were harvested and subjected to quantitative real-time polymerase chain reaction (qRT-PCR) or western blotting. The primers for miR-199b-5p, anti-miR-199b-5p and NC are listed in Supplementary Table S1.

To establish stable overexpression of DDR1 in PCa cells, full-length DDR1 was designed by GenePharma (Shanghai, China) and cloned into pcDNA3.1 (t) expression vector (Invitrogen, Carlsbad, CA, USA). pcDNA3.1- DDR1 was transfected into cells as mentioned above. For establishing stable knockdown of DDR1 in PCa cells, lentiviral vectors labelled Firefly Luciferase, which contain a specific shRNA (a total of 4 shRNAs in this study, named as shRNA #1, shRNA#2, shRNA#3 and shRNA#4, respectively) against DDR1 (LV16-shRNA-DDR1) were designed based upon Oligo Designer 3.0. The sense strand was 5’-T-(GN18)-(TTCAAGAGA)-(N18C)-TTTTTTC-3’ and the antisense strand was 3’-A(CN18)-(AAGTTCTCT)-(N18G)-AAAAAAG AGCT-5’. Briefly, LV16-shRNA-DDR1 was constructed successively through XhoI enzymes restriction, shDNA annealing (95 °C 5 min; 85 °C 5 min; 75 °C 5 min; 70 °C 5 min; 4 °C), linearisation, endonuclease reaction, extraction (Agarose Gel DNA Purification Kit Ver2.0), ligation reaction, transferred to competent recipient cells, and lentivirus package. Lentivirus titration was conducted by transducing 293 T cells, and the multiplicity of infection (MOI) was 2–5 × 10^8^ TU/ml. Cells were infected with the lentivirus in the presence of 5 μg/ml Polybrene and the transfected cells were screened by 1 μg/ml Puromycin (Clontech, USA) following the manufacturer’s instructions. The expression of DDR1 was determined by qRT-PCR and western blotting.

### Cell proliferation, migration and invasion assays

The effects of miR-199b-5p on the proliferation ability of PCa cells were measured by 3-(4,5-dimethylthiazol-2-yl)-5-(3-carboxymethoxyphenyl)-2-(4-sulfophenyl)-2H-tetrazolium (MTS) assay (MTS, Promega Corporation, Fitchburg, WI) and colony formation assay. Wound-healing assay measuring gap distance and transwell chambers were used to assess the capability of cell migration and invasion, respectively. All these procedures were conducted as described previously by our group.^12–14^ Briefly, NC and transfected PCa cells were seeded in a 96-well plate at 2000 cells/well. At the indicated time points (days), cell viability was measured by adding 10 μL MTS to the medium and incubating at 37 °C for 4 h prior to spectrophotometrically reading absorbance at 490 nm by using a multiplate reader (Bio Tek Instruments, Inc. USA). For colony formation assay, PCa cells were seeded in six-well plates at a density of 1 × 10^3^ cells/well. After 1-2 weeks, colonies of >50 cells were scored after being visualised by fixing with 10% formaldehyde and staining with 1% crystal violet. The photographs of colonies growing in the plates were taken. For wound-healing assay, PCa cells were plated in 6-well plates and cultured until 80% confluence. A wound was produced by manually scraping the cell monolayer with a 10 μL sterile pipette tip. Cells were washed by PBS and then cultured with fresh serum-free medium. Cell migration was observed, and images of the scratches were filmed under the optical microscope (CKX41, Olympus) at the indicated time points (hours). Transwell invasion assay was performed in 24‐well cell culture chambers using transwell inserts with 8 μm pore size (Corning Incorporated, Corning, NY, USA). The membranes of transwell inserts were coated with Matrigel (BD Biosciences). A total of 5 × 10^4^ cells was resuspended in 100 μL serum-free medium and plated into upper chamber. RPMI-1640 containing 10% FBS was added to the lower chamber. After incubation for 48 h, cells on upper surface of the insets were removed with a cotton swab, while cells on lower surface were immobilised with 4% paraformaldehyde and then stained with 0.1% crystal violet for 15 min. Invasive cells were viewed and counted under the inverted microscope.

### miR-199b-5p target gene prediction and luciferase reporter assay

Prediction of miR-199b-5p target genes was accomplished by using TargetScan (http://www.targetscan.org/), miRDB (http://mirdb.org/), miRWalk (http://zmf.umm.uni-heidelberg.de/) and miRTarBase (http://mirtarbase.mbc.nctu.edu/) online tools. To corroborate the predicted targets, the expression of the target genes was detected in transfected PCa cells with miR-199b-5p, anti-miR-199b-5p and NC by qRT-PCR and western blotting. To further verify the target sites of miR-199b-5p, dual luciferase reporter assay was carried out. Full-length 3′-UTR sequence of DDR1 and that of a target-site mutant were designed and obtained from GenePharma (Shanghai, China), amplified by PCR, and cloned into the pLUC-REPORT luciferase expression vector (Ambion, Austin, TX, USA) as pLUC-DDR1-3’UTR- wild type (WT) and pLUC-DDR1-3’UTR- mutant type (Mut), respectively. A total of 2 × 10^5^ LNCaP, C4-2, and PC-3 cells was seeded in each well of 24-well plates, and co-transfected with miR-199b-5p or anti-miR-199b-5p and pLUC-DDR1-3’UTR-WT and pLUC-DDR1-3’UTR- Mut using Lipofectamine 3000 (Invitrogen, Thermo Fisher Scientific, Inc.). Forty-eight hour later, cells were collected and the relative luciferase activity in each group was measured by Dual Luciferase Reporter Assay System kit (Promega, Madison, WI, USA) on a Centro LB960 XS3 (Berthold, German) following manufacturer’s instructions. Firefly luciferase activity was used for normalisation.

### Bioinformatics analyses

In this study, we used the Taylor dataset,^15^ a publicly available dataset, to compare the expression levels of miR-199b-5p in tissue specimens from normal prostate, primarily localised and metastatic PCa patients. Moreover, to explore the expression pattern and clinical significance of DDR1 in PCa, The Cancer Genome Atlas database (TCGA, https://tcgadata.nci.nih.gov/tcga/) with the online tool Gene Expression Profiling Interactive Analysis (GEPIA, http://gepia.cancer-pku.cn/) and the Gene Expression Omnibus (GEO, http://www.ncbi.nlm.nih.gov/geo/) database with accession number: GSE16560 were utilised to analyse the expression of DDR1 in normal prostate and PCa tissues.

### Dual-colour immunofluorescence assays

A two-step dual-colour immunofluorescence in situ hybridisation (FISH) was performed to detect the in situ co-expression of miR-199b-5p and DDR1 on formalin-fixed paraffin-embedded 4-μm thick sections from clinical PCa tissue samples. Firstly, after deparaffinisation and dehydration, the tissue sections were hybridised with miR-199b-5p detection probe labelled with green fluorescence (Servicebio, Wuhan, China) by using FISH kit (Bosterbio, USA) according to the manufacturer’s protocol. Secondly, the tissue sections were incubated with the primary antibody of DDR1. Alexa Fluor^®^ goat-anti-rabbit antibody with red fluorescence (Invitrogen, USA) was applied as the secondary antibody. Finally, the slides were washed with wash buffer followed by counterstaining with 4‐6‐diamidino‐2‐phenylindole (DAPI, Servicebio, G1012).

In addition, we used the dual-colour immunofluorescence (IF) staining to detect the co-expression of EMT markers in PCa cells and xenograft tumours samples. For PCa cells, LNCaP and PC-3 cells were grown on poly-L-lysine-coated slides (Nalge Nunc, Rochester, NY), respectively. When the cells reached 80% confluence, the slides were washed in PBS and then fixed with 4% paraformaldehyde for 10 min min at room temperature. To reduce nonspecific staining, each section was blocked with 2% FBS in PBS for 30 min. For xenograft tumour tissues, the sections were processed following the immunohistochemical (IHC) protocol. The slides were incubated with primary antibodies against mouse monoclonal anti-E-cadherin (green fluorescence, 1:50 for cells and 1:250 for tissues, Proteintech 60335-1-ig) and rabbit polyclonal anti- vimentin (red fluorescence, 1:300 for cells and 1: 50 for tissues, Servicebio GB11192) or rabbit monoclonal anti-fibronectin (red fluorescence, 1:200 for cells and 1: 50 for tissues, Servicebio GB13091) overnight at 4 °C in a humidity chamber. Then, the sections were incubated with CY3-conjugated goat anti-rabbit IgG (Servicebio GB21303) and FITC-conjugated goat anti-mouse IgG (Servicebio GB22301) secondary antibodies at 1:100 for 1 h. Nuclei were stained with DAPI before mounting coverslip with vector shield medium.

All sections were visualised on a fluorescent microscope (AX80, Olympus, Tokyo, Japan). Five random microscope fields per section were recorded and the integral optical density of every visual field was calculated.

### Animal experiments

Ninety male BALB/c-nu/nu athymic nude mice, 5 weeks old and body weights 16–18 g on arrival, were purchased from Experimental Animal Center of Guangdong Province (Guangzhou, China). The mice were maintained in a special pathogen free facility and housed in wire-top plastic cages with five per cage and kept in an environmentally controlled room (25 °C temperature, 50% humidity and lit 12 h/day) with free access to fresh water and solid pellet diet. All procedures related to the animal experiments were carried out in accordance with a mouse protocol, which was reviewed and approved by the Institutional Animal Care and Use Committee of the First Affiliated Hospital of Guangzhou Medical University.

To evaluate the effects of miR-199b-5p on tumour growth, a concentration of 5 × 10^6^ cells/ml, suspended in a 0.1 ml 1:1 mixture of culture medium and Matrigel on ice, were inoculated subcutaneously in the flank region of the mice to establish xenograft tumours. The animals were individually ear-marked when the cancer cells were inoculated. Forty-five mice were randomly assigned to nine experimental groups of five animals each: LNCaP- miR-199b-5p, LNCaP-anti- miR-199b-5p, LNCaP-NC; C4-2- miR-199b-5p, C4-2-anti-miR-199b-5p, C4-2-NC; PC-3-miR-199b-5p, PC-3-anti-miR-199b-5p and PC-3-NC. After the subcutaneous inoculation of the cancer cells, the mice were examined daily for signs of tumour formation, and the tumour sizes were monitored twice weekly by calliper measurements of length and width. This procedure was done by two individuals (SZ and LL) in various combinations, one to restrain the mouse and the other to make the tumour measurements. The tumour volumes were calculated using the formula: volume (in mm^3^) = length × width^2^ (in mm)/2. For each group, the tumour volume was calculated as the mean value±standard deviation (SD). At 4 weeks after injection, the mice were sacrificed by cervical dislocation under isoflurane anaesthesia, and the xenograft tumours were harvested and divided into two halves. One piece was flash-frozen in liquid nitrogen and stored at −80 °C for mRNA or protein extraction, and the other was fixed in neutral buffered formaldehyde (10% w/v) and embedded in paraffin wax for histological examination and immunohistochemical assay.

For the tumour metastasis experiments, imaging of living mice was performed. The nine cell models mentioned above were transfected with firefly luciferase vector, and then injected into five mice each group at a concentration of 2 × 10^6^ cells for each mouse via the tail vein. After anesthetised with 2% isoflurane in oxygen at 2 l/min and intraperitoneally administered with D-luciferin (3 mg in 100 µl PBS per mouse), the mice were imaged weekly in the Xenogen IVIS Spectrum Imaging System (PerkinElmer) under sequence luminescence scan mode using 5–300 sec acquisitions to measure FL bioluminescence activity. Living Image 3.2 software (PerkinElmer) was used to quantify tumour FL activity in terms of average radiance (photon/s/cm^2^/sr).

### RNA extraction and qRT-PCR

Total RNAs from cells, xenograft tumours and clinical PCa tissues were isolated using Trizol reagent (Invitrogen). RNA was reversely transcribed into first-strand cDNA with All-in-One First-Strand cDNA Synthesis kit (GeneCopoeia, Guangzhou, China). For miR-199b-5p detection, TaqMan MicroRNA Reverse Transcription kit (Thermo Fisher Scientific Inc., Waltham, MA, USA) and sequence specific primers (GeneCopoeia, Guangzhou, China) were used. qPCR for quantification of gene or miRNA expression was performed with the TB Green Premix Ex Taq kit (Takara, Toyoko, Japan) on a CFX96 system (Bio-Rad, Hercules, CA). GAPDH and U6 RNA were used as endogenous controls for genes and miRNA, respectively. Relative expression analysis was performed by using the 2^−△△CT^ method. All experiments were carried out in triplicate. The sequences of the primers used in this study were listed in Supplementary Table S1.

### Protein extraction and western blotting

Total proteins were extracted from cells and xenograft tumour tissues, and western blotting assay was carried out as described previously by our group.^16,17^ Briefly, the obtained proteins were separated by 10% SDS‐PAGE electrophoresis and then transferred onto PVDF membranes (Invitrogen). The membranes were blocked in 5% defatted milk for 2 h and incubated overnight with primary antibodies at 4 °C, followed by horseradish peroxidase (HRP)-conjugated secondary antibodies against mouse and rabbit IgG (Sigma–Aldrich, St. Louis, MI) for 1 h at room temperature. The immunoblots were detected by LI‐COR Odyssey^Ⓡ^ CLX Two‐colour infrared laser imaging system (LI‐COR Biosciences). Densitometric analysis of the bands was implemented using ImageJ software (National Institutes of Health). Primary antibodies against AR, DDR1, total ERK, phosphorylate-ERK (p-ERK), E-cadherin, vimentin, fibronectin and GAPDH (internal reference) were obtained from Abcam (Cambridge, MA).

### Histological and immunohistochemical assessment

Paraffin-embedded tissues of mouse-derived tumours and clinical PCa were cut into 4-μm sections for haematoxylin and eosin (HE) staining and immunohistochemical (IHC) staining. HE staining was used to assess tumour morphology. IHC analysis was performed as described previously by our group.^17,18^ Briefly, after deparaffinisation, rehydration, antigen retrieval, and blocking endogenous peroxidase activity, the sections were incubated with the primary antibody mouse anti‐DDR1 (Abcam, Cambridge, MA) overnight at 4 °C, followed by adding the secondary biotinylated antibody in PBS for 20 min at room temperature. Finally, sections were treated with streptavidin-HRP in PBS for 30 min at room temperature, and subsequently incubated with diaminobenzidine buffer (Vector Laboratories, Burlingame, CA). The negative

control used PBS to substitute for primary antibody. Sections were counterstained with haematoxylin, rinsed with water and dehydrated through ethanol to xylene and mounted with DPX (Sigma–Aldrich Company Ltd). The DDR1 expression level was evaluated by assessing positive cell percentages and staining intensities according to the protocol described previously by our group.^14^ The relative quantification of protein expression was implemented by using Image‐Pro Plus 6.0 (Media Cybernetics).

### Statistical analyses

Statistical analyses in this study were performed by using the SPSS 18.0 software (SPSS, Inc., Chicago, IL). All data were presented as mean ± SD from at least three

independent experiments. Two groups were compared with two-tailed Student’s *t*-test. One-way analysis of variance followed by Tukey’s test was applied to explore the differences among multiple groups. The correlation between miR-199b-5p and DDR1 expression levels was estimated by Pearson’s correlation coefficient analysis. The correlations between miR-199b-5p expression and clinicopathological characteristics of PCa patients were analysed using Mann–Whitney U and Kruskal–Wallis H tests. Survival analyses were calculated by the Kaplan–Meier method and compared between the groups using the log-rank test. *P* < 0.05 was considered statistically significant.

## Results

### miR-199b-5p is significantly downregulated in metastatic PCa tissues and cells, independently of AR expression

**Fig. 1:**
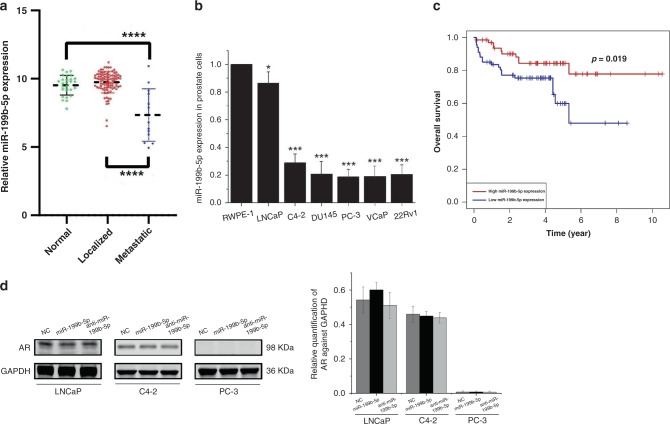
miR-199b-5p expression is remarkably downregulated in metastatic PCa tissue samples and cells, but independent of AR expression. **a** The expression levels of miR-199b-5p in tissue specimens of the normal prostate (*n* = 28), and the localised (*n* = 94) and metastatic PCa patients (*n* = 19), as from microarray analysis in Taylor dataset. **b** qRT-PCR assay verified a remarkably downregulated level of miR-199b-5p expression in the metastatic, castration-resistant PCa cells (VCaP, C4-2, DU145, 22RV1 and PC-3), as compared with that in the normal prostate epithelial cell (RWPE-1) and weakly metastatic, androgen-dependent PCa cell (LNCaP). U6 was used as endogenous control. **c** Kaplan–Meier analysis from Taylor dataset showed a significantly shorter overall survival in PCa patients with low miR-199b-5p levels than those with high miR-199b-5p levels. **d** Western blotting showed that AR was not involved in miR-199-5p expression in PCa cells. *P < 0.05, ***P < 0.001 and ****P < 0.0001.

By analysing miR-199b-5p expression levels in Taylor dataset, we observed that miR-199b-5p was significantly downregulated in tumour tissues from metastatic PCa patients (*n* = 19) when compared to those from normal prostate (*n* = 28, *P* < 0.0001) and localised PCa patients (*n* = 94, *P* < 0.0001) (Fig. 1a). We further corroborate the expression of miR-199b-5p in normal prostate epithelial cell RWPE-1 and PCa cells LNCaP, C4-2, VCaP, 22Rv1 and PC-3 by using qRT-PCR assay. As shown in Fig. 1b, the expression levels of miR-199b-5p was significantly lower in metastatic, castration-resistant VCaP, C4-2, 22Rv1 and PC-3 cells than that in normal RWPE-1 cell and weakly metastatic, androgen-dependent LNCaP cell (*P* < 0.05 for all). Moreover, Kaplan–Meier analysis revealed that PCa patients with low miR-199b-5p levels had a significantly shorter overall survival (*P* = 0.019) than those with high miR-199b-5p levels (Fig. 1c). These findings suggested that miR-199b-5p functions as a tumour suppressor in PCa metastasis.

Androgen deprivation therapy (ADT) is a first line of therapy for metastatic PCa, and androgen receptor (AR) is actively involved in the castration resistance of PCa. In this study, to verify whether AR is involved in miR-199-5p expression in PCa cells, we primarily performed in vitro gain-of-function and loss-of-function experiments in LNCaP, C4-2, and PC-3 cells by stably transfecting these cells with miR-199b-5p and anti-miR-199b-5p. As illustrated in Fig. 1d, the results of western blotting showed that there were no significant difference in the expression level of AR among AR-positive LNCaP and C4-2 cells, AR-negative PC-3 cells and their miR-199b-5p and anti-miR-199b-5p-transfected counterparts, respectively (*P* < 0.05 for all), suggesting that miR-199b-5p has no effects on AR expression in PCa cells.

### miR-199b-5p suppresses proliferation, migration and invasion of PCa cells

In this study, we used in vitro gain-of-function and loss-of-function experiments to determine the role of miR-199b-5p in the proliferation, migration and invasion of PCa cells. The qRT-PCR analysis confirmed the marked overexpression of miR-199b-5p in the miR-199b-5p infected cells compared with that in control cells (all *P* < 0.01, Supplementary Fig. S1A). MTS and colony formation assays showed that miR-199b-5p-transfected cells were markedly reduced in number of living cells and colony formation when compared to the anti-miR-199b-5p and NC-transfected counterparts (*P* < 0.05 for all, Supplementary Fig. S1 B, C), indicating a significantly inhibited growth of PCa cells by upregulation of miR-199b-5p. Moreover, wound-healing assay, and transwell migration and invasion assays demonstrated that miR-199b-5p significantly decreased the migratory and invasive capacities of these cells, while anti-miR-199b-5p dramatically facilitated the capacity of cell migration and invasion (*P* < 0.05 for all, Supplementary Fig. S1 D, E).

### miR-199b-5p suppresses tumour growth and metastasis in vivo

**Fig. 2:**
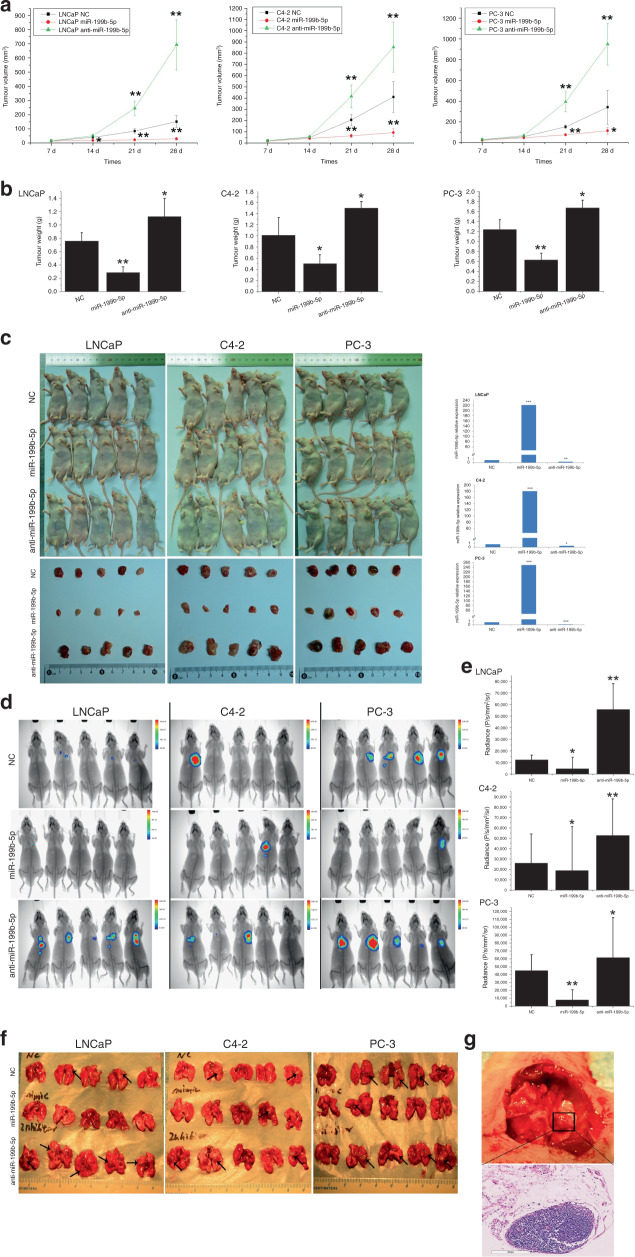
miR-199b-5p inhibits tumour growth and metastasis in vivo. **a** Graphs showed the tumour growth volume of xenografts derived from miR-199b-5p, anti-miR-199b-5p and NC-transfected PCa cells (5 mice per group), as measured with a calliper at the indicated days after injecting these cells into male BALB/c-nu/nu athymic nude mice. **b** Bar plots showed the tumour weight of xenografts originated from the mice inoculated with these transfected cells. **c** Four weeks after implantation, the tumours were excised and imaged. The representative appearance of tumour mass resected from each group of mice at autopsy on day 28 after subcutaneous injection (left). qRT-PCR showed relative expression of miR-199b-5p in xenografts derived from miR-199b-5p and anti-miR-199b-5p-transfected cells as compared to those derived from NC cells, at 28 days after injection into mice. U6 was used as the endogenous control (right). **d** The metastatic lesions in the mice were monitored by bioluminescence imaging at 4 weeks after tail vein injection. **e** FL bioluminescence activity of mice bearing metastatic tumours (in **d**) was quantified in terms of average radiance (photon/s/cm^2^/sr) using the Living Image 3.2 software. **f** The sites of metastatic lesions in mice shown in **d** were further confirmed by autopsy analysis and the metastatic tumours were subsequently whole excised. The results showed that the organs involved in metastases were mostly the lungs. **g** Representative images of lung metastasis at autopsy (top) with the histological confirmation by HE staining (bottom). Error bars represent the mean ± SD of three independent experiments. *P < 0.05 and **P < 0.01.

To elucidate the effects of miR-199b-5p on invasive and metastatic features of PCa in vivo, subcutaneous tumour xenograft model and metastasis experimental model were established by injecting miR-199b-5p, anti-miR-199b-5p and NC-transfected PCa cells into the flank or tail vein of male BALB/c-nu/nu athymic nude mice, respectively. In the study, because we observed that significant differences in cells growth began to emerge at 3 days after transfection, the miR-199b-5p expression levels were detected in the transfected PCa cells at this time-point (Supplementary Fig. S1A). As shown in Supplementary Fig. S1, although the expression levels of miR-199b-5p were significantly higher in miR-199b-5p-transfected LNCaP, C4-2 and PC-3 cell than these in NC cells, insignificantly reduced miR-199b-5p levels were observed in cells by anti-miR-199b-5p transfection as compared to NC cells. Thus, in this study, to ensure that the injected PCa cells had stably expressed miR-199b-5p or downregulated miR-199b-5p for sufficient time to impact on xenograft growth, the stability and functionality of the miR-199b-5p and anti-miR-199b-5b transfected LNCaP, C4-2 and PC-3 cells were evaluated in vitro and in vivo. As shown in Supplementary Fig. S2, the results of qRT-PCR analyses verified in vitro that miR-199b-5p-transfected LNCaP, C4-2 and PC-3 cells maintained significantly higher expression levels of miR-199b-5p than NC cells for at least 21 days (all *P* < 0.001) while anti-miR-199b-5p-transfected cells had significantly reduced miR-199b-5p levels after 7 days for transfected LNCaP and PC-3 cells, and after 14 days for transfected C4-2 cells as compared to NC cells, respectively (all *P* < 0.05). Functionally, in line with the cell growth data in vitro, miR-199b-5p supplementation resulted in dramatically slower tumour growth in vivo, while downregulation of miR-199b-5p expression significantly promoted tumour formation and growth (*P* < 0.05 for all, Fig. 2a–c). Moreover, in line with in vitro data, Fig. 2c showed that at 28 days after injection into mice, miR-199b-5p was still about 210-, 180- and 250-fold more expressed in the xenografts derived from miR-199b-5p-transfected LNCaP, C4-2 and PC-3 cells as compared to NC ones, respectively (all *P* < 0.001), whereas xenografts derived from anti-miR-199b-5p-transfected cells maintained significantly lower levels of miR-199b-5p than NC ones (all *P* < 0.05). Furthermore, results from in vivo bioluminescence imaging of mice bearing metastatic tumours demonstrated that miR-199b-5p could significantly suppress tumour metastatic growth at 4 weeks following tail vein injection, which revealed the markedly reduced FL average radiance derived from LNCaP- miR-199b-5p, C4-2- miR-199b-5p and PC-3- miR-199b-5p cells when compared with anti-miR-199b-5p cell and NC cell (Fig. 2d, e, *P* < 0.05 for all). In addition, we found that knockdown of miR-199b-5p expression remarkably prompted lung metastasis formation in LNCaP cell (Fig. 2f, g, *P* = 0.004). Taken together, these results provide evidence about miR-199b-5p involving in the inhibition of PCa growth and metastasis. In this study, although the mice injected with C4-2- miR-199b-5p cells presented the lower FL activity than these with anti-miR-199b-5p cell and NC cell (Fig. 2d), the number of FL expression focus in these three groups of mice was insignificantly different. Therefore, we chose LNCaP and PC-3 cells in the subsequent study of the mechanism.

### Discoid domain receptor 1(DDR1) is a functional target of miR-199b-5p in PCa

**Fig. 3:**
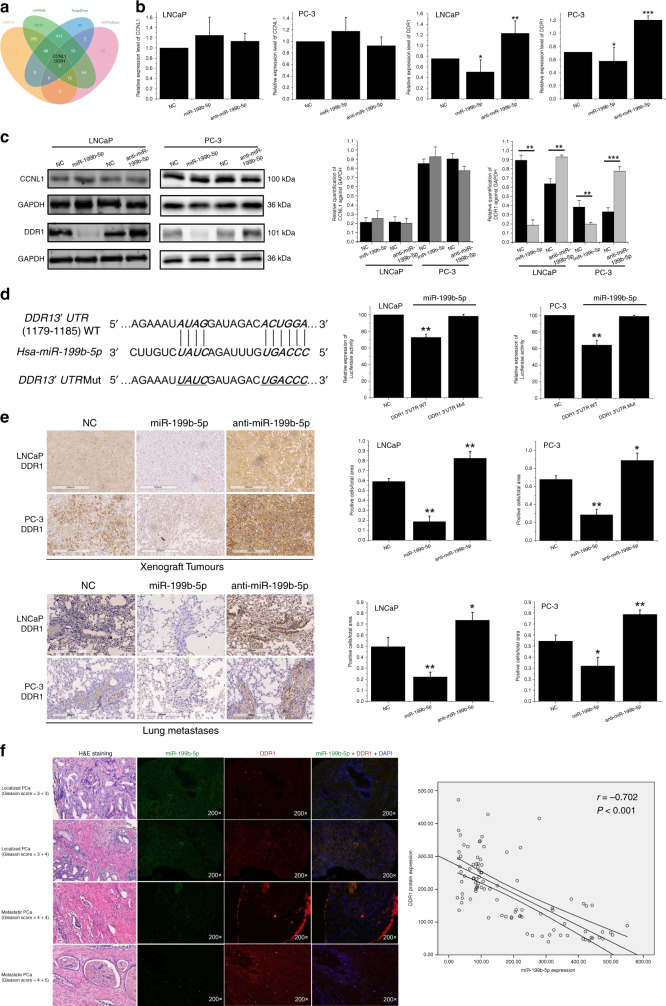
DDR1 is a functional target of miR-199b-5p in PCa. **a** Venn diagram showed the overlap from TargetScan, miRDB, miRWalk and miRTarBase, identifying CCNL1 and DDR1 as potential targets of miR-199b-5p. **b** qRT-PCR assay showed a significantly downregulated expression level of DDR1 mRNA in miR-199b-5p-transfected LNCaP and PC-3 cells as compared to anti-miR-199b-5p and NC-transfected cells (right), but no changes of CCNL1 mRNA expression detected in these transfected PCa cells (left). U6 was used as the endogenous control. **c** Western blotting was carried out to verify the expression of CCNL1 and DDR1 at protein level in these transfected PCa cells, showing the similar results with **b**. GAPDH was used as the endogenous control. **d** Schematic diagram exhibited consequential pairing of miR-199b-5p and DDR1 mRNA 3’-UTR, as from miRanda database (http://www.microRNA.org) (left). Mutant-3’UTR type (Mut) indicated the 3’-UTR of DDR1 mRNA with the targeted mutation in miR-199b-5p putative binding sites (left). Luciferase reporter assay confirmed the markedly suppressed luciferase activity of DDR1 3’UTR-luc-WT in miR-199b-5p-transfected LNCaP and PC-3 cells, but the luciferase expression of DDR1 3’UTR-luc-Mut was not changed (right). **e** IHC was performed to evaluate DDR1 expression levels in the tissues of tumour xenografts (top) and lung metastases (bottom) derived from the mice injected with miR-199b-5p, anti-miR-199b-5p and NC-transfected LNCaP and PC-3 cells (left). Scale bar, 200 µm. Bar plots showed a negative correlation between miR-199b-5p and DDR1 expression levels by quantifying the number of positive staining cells in IHC slides (right). **f** Dual-colour FISH confirmed that downregulation of miR-199b-5p is inversely correlated with DDR1 upregulation in clinical PCa (Pearson’s correlation r = −0.702, *P* < 0.001). ×200. Error bars represent the mean ± SD of three independent experiments. *P < 0.05, **P < 0.01 and ***P < 0.001.

To understand the mechanisms underlying the tumour-suppressive role of miR-199b-5p in PCa, the miRNA targets search was performed by using TargetScan, miRDB, miRWalk and miRTarBase in this study. The results showed Cyclin L1 (CCNL1) and DDR1 as the candidate targets of miR-199b-5p (Fig. 3a). Then, we evaluated the expression levels of CCNL1 and DDR1 in miR-199b-5p, anti-miR-199b-5p and NC-transfected LNCaP and PC-3 cells using qRT-PCR and western blotting assays. The results revealed that miR-199b-5p induced a significant decline of DDR1 mRNA and protein expression (*P* < 0.05 for all) but had no effects on CCNL1 expression (*P* > 0.05 for all, Fig. 3b, c). Moreover, as shown in Fig. 3b, c, the expression levels of DDR1 mRNA and protein were decreased in miR-199b-5p overexpressed PCa cells while they were increased in miR-199b-5p downregulated cells (*P* < 0.05 for all). These findings indicated a negative correlation between miR-199b-5p and DDR1 expression, thus prompting us to validate DDR1 as a potential target of miR-199b-5p in PCa cells. One site complementary to the seed sequence of mature miR-199b-5p was observed in position from 1179 to 1185 of DDR1 mRNA 3’-UTR region (Fig. 3d). To further determine whether miR-199-5p directly regulates DDR1, luciferase reporter assay was carried out in LNCaP and PC-3 cells. The results demonstrated that miR-199b-5p markedly suppressed the luciferase activity of DDR1 3’UTR-luc-WT in LNCaP and PC-3 cells (*P* < 0.01 for all), but had no effects on the luciferase expression of DDR1 3’UTR-luc-Mut in these cells (*P* = 0.238 and *P* = 0.315, respectively) (Fig. 3d). These results indicate that DDR1 is the direct functional target of miR-199b-5p.

For corroborating these findings, we performed IHC to evaluate the expression levels of DDR1 protein in the tumour xenografts and metastatic tumours tissues derived from the mice injected with miR-199b-5p, anti-iR-199b-5p and NC-transfected LNCaP and PC-3 cells. The results showed that the protein levels of DDR1 were significantly downregulated in subcutaneous tumour xenografts and lung metastases derived from miR-199b-5p-transfected cells, as compared to NC group (*P* = 0.003 for LNCaP cell group and *P* = 0.008 for PC-3 cell group, respectively), whereas the remarkably upregulated DDR1 expression levels were observed in the group from anti-miR-199b-5p-transfected cells (*P* = 0.006 for LNCaP cell group and *P* = 0.02 for PC-3 cell group, respectively) (Fig. 3e). These findings revealed that miR-199b-5p negatively regulated DDR1 expression in vivo.

Furthermore, we investigated miR-199b-5p and DDR1 expression in clinical PCa tissues by using dual-colour immunofluorescence in situ hybridisation (FISH). As illustrated in Fig. 3f and Table 1, the expression levels of miR-199b-5p (green fluorescence) were attenuated with the increase of Gleason scores and the lowest in metastatic PCa, while the corresponding DDR1 expression levels (red fluorescence) in the same tissue section were enhanced with Gleason score increases and highest in metastatic disease. The results of Pearson’s correlation coefficient analysis corroborated that downregulation of miR-199b-5p is inversely correlated with DDR1 upregulation in PCa tissues (r = −0.702, *P* < 0.001). These results demonstrated the involvement of miR-199b-5p/DDR1 in the clinical progression of PCa. Overall, these evidences support the hypothesis that tumour-suppressive effects of miR-199b-5p are ascribable to its ability to directly repress DDR1.

**Table 1:** Clinicopathological characteristics of PCa patients.

Variables	Total	DDR1 (+)	DDR1 (−)	*P*	miR-199b-5p (+)	miR-199b-5p (−)	*P*
Patients (no, %)	94	66 (70.2%	28 (29.8%)		37 (39.4%)	57 (60.6%)	
Age (year)							
≤65	58	38	20	0.206	24	34	0.424
>65	36	28	8		13	23	
PSA level at diagnosis (ng/mL)							
<10	27	21	6	0.184	8	19	0.061
10–20	30	23	7		17	13	
>20	37	22	15		12	25	
Gleason score							
<7	38	11	27	<0.001	17	21	<0.001
=7	28	22	6		18	10	
>7	28	17	11		2	26	
PT stage							
≤T2	65	41	24	0.024	27	38	0.278
≥T3	29	25	4		10	19	
Lymph node involvement							
Negative	89	62	27	0.623	34	55	0.636
Positive	5	4	1		2	3	
Distal metastases							
Yes	38	38	0	<0.001	8	30	0.003
No	56	28	28		29	27	

### DDR1 reverses the suppressive effects of miR-199b-5p in PCa cells

To verify that miR-199b-5p inhibits PCa cell proliferation, migration and invasion by downregulating DDR1, we performed rescue experiments. Based on the expression levels of DDR1 mRNA and protein in LNCaP and PC-3 cells, which were shown in Supplementary Fig. S3A, we successfully upregulated DDR1 expression in LNCaP cells by transfecting pcDNA3.1- DDR1 (Supplementary Fig. S3B), and knocked down the expression of DDR1 in PC-3 cells by transfecting LV16-shRNA-DDR1 (shRNA#1 or shRNA#2) (Supplementary Fig. S3C). MTS, colony formation, wound-healing and transwell assays revealed that RNAi (shRNA#1 and shRNA#2)-mediated DDR1 knockdown could dramatically suppress PC-3 cell proliferation, migration and invasion (*P* < 0.05 for all, Supplementary Fig. S3D–G). In this study, we conducted “wound-healing” to analyse the changes of cells migration rates, which was performed by manually scraping the cell monolayer with pipette tips. As shown in Supplementary S1D, the migration rates of LNCaP-NC and PC-3 NC cells are almost the same (45% vs 50%), while in Supplementary Fig. S3F, the migration rates of LNCaP vector and PC-3 scramble shRNA are 18% vs 64%. This difference might be caused by the inconsistent “wound” width of each hand scratch. However, these cells were all used as controls for intra-group comparison in the study. Therefore, we can assume that this difference could not amplify the effect of pcDNA3.1- DDR1 and shRNA-DDR1 on cell migration ability. Nevertheless, this might be one limitation of the present study. Consistent with the evidence that DDR1 is a functional target of miR-199b-5p, these results also showed that DDR1 silencing by shRNA phenocopied the inhibited growth, migration and invasion of miR-199b-5p-transfected cells. Moreover, co-transfection of miR-199b-5p and pcDNA3.1-DDR1 in both LNCaP and PC-3 cells could attenuate the suppressive effects of miR-199b-5p on the proliferative, migratory and invasive capabilities of these cells (*P* < 0.05 for all) (Supplementary Fig. S3H–K). These data indicated that DDR1 restoration could reverse the inhibitive effects of miR-199b-5p on PCa cells at least in part.

### DDR1 affects ERK signalling pathway and induces epithelial-mesenchymal transition (EMT) phenotype in PCa

**Fig. 4:**
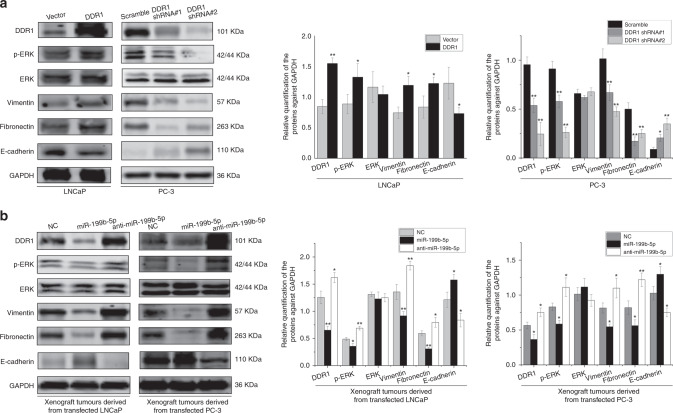
DDR1 affects ERK signalling pathway in PCa. **a**, **b** Western blotting assay showed the effects of DDR1 upregulation and downregulation on the expression levels of total ERK, p-ERK and EMT-related markers in LNCaP and PC-3 cells (**a**), and the effects of miR-199b-5p restoration on the expression levels of DDR1, total ERK, p-ERK and EMT-related markers in xenograft tumours (**b**). *P < 0.05 and **P < 0.01.

DDR1 overexpression is reported to be associated with EMT,^19^ and DDR1 as a non-integrin collagen receptor could mediate the extracellular signal-regulated kinase (ERK) pathway. It is well-known that EMT promoted the invasion and metastasis of cancer cells. Additionally, ERK signalling pathway is known to have an important role in the regulation of cell growth, EMT and metastasis.^20–22^ In this regard, we evaluated the expression amount of total ERK and its active form phosphorylate-ERK (p-ERK) at protein level in PCa cells. The results from western blotting showed that p-ERK expression level was significantly enhanced in DDR1 overexpressed LNCaP cells whereas it was decreased in DDR1 silenced PC-3 cell as compared to the NC cells (*P* < 0.001 for all, Fig. 4a), but there were no significant differences in expression level of the total ERK in these cells. Furthermore, we assessed the total ERK and p-ERK expression levels upon miR-199b-5p supplementation in xenograft tumours derived from the mice injected with LNCaP and PC-3 cell models, and found that p-ERK was significantly downregulated at protein level in tumours from miR-199b-5p-transfected PCa cell model while it was upregulated in those from anti-miR-199b-5p-transfected cell model (*P* < 0.001 for all, Fig. 4b), but there were no significant change in expression level of the total ERK in tumour tissues from these cells.

**Fig. 5:**
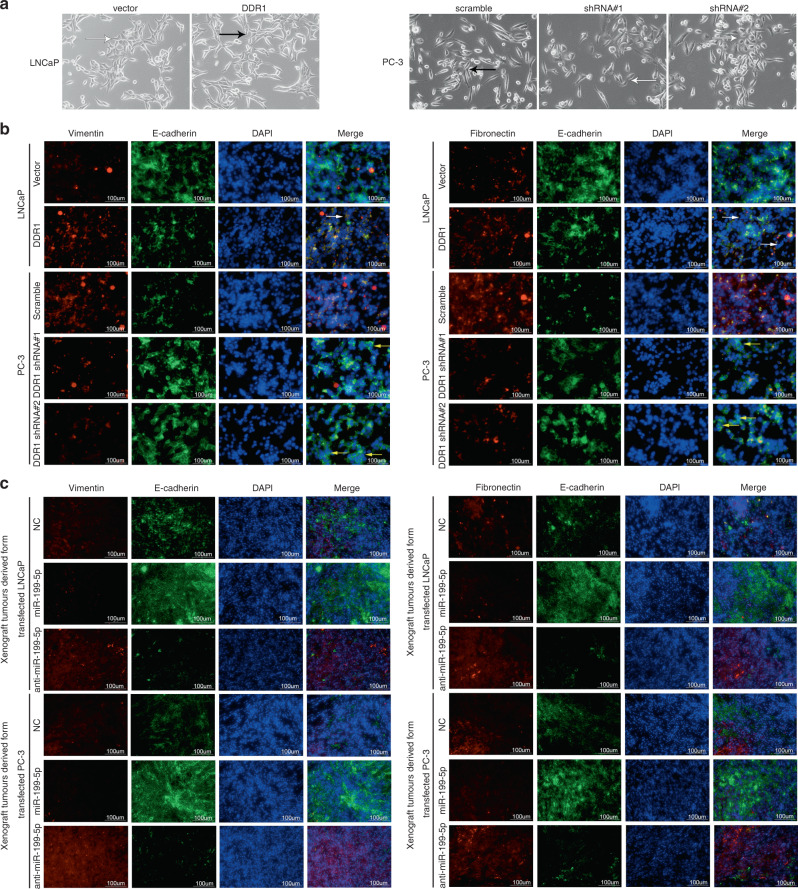
DDR1 induces EMT phenotype in PCa. **a** Representative bright-field microphotographs showed the effects of DDR1 upregulation and downregulation on the morphological changes occurring in LNCaP and PC-3 cells. The white arrowhead indicated a morphological appearance of cobblestone-like epithelial cell, and the black one indicated a morphological appearance of fibroblast-like mesenchymal cell. **b**, **c** Representative immunofluorescence microphotographs for dual-colour IF staining showed the co-expression of EMT markers in PCa cells (**b**) and xenograft tumours from these cells (**c**).

As shown in Fig. 4a, we observed the overexpression of the mesenchymal markers, vimentin and fibronectin and the downregulation of the epithelial marker E-cadherin expression at protein level in the DDR1-overexpressed PCa cells. Also, these cells lost epithelial morphological features and presented a fibroblast-like mesenchymal appearance (Fig. 5a), suggesting the occurrence of EMT. These cells became more disperse compared to the NC cells, indicating the enhanced motility. This complies with the evidence that these cells have the promoted ability to form metastatic nodes in vivo (Fig. 2d–f). EMT is characterised by a spindle-like morphology of tumour cells and gain of mesenchymal markers. To accommodate the phenotypic changes, we conducted the dual-colour IF staining to detect the co-expression of EMT markers in vitro and in vivo. The results of dual-colour IF staining in PCa cells corroborated that DDR1-overexpressed LNCaP and PC-3 cells were E-cadherin negative and gained positive expression for vimentin and fibronectin (Fig. 5b). Moreover, the miR-199b-5p expression was remarkably reduced in the xenograft tumours, along with the downregulated expression of E-cadherin and the upregulated expression of DDR1, vimentin and fibronectin (Fig. 4b). The dual-colour IF staining also verified that the decreased E-cadherin expression but increased vimentin and fibronectin expression were observed in xenograft tumours derived from the anti-miR-199b-5p-transfected PCa cell model (Fig. 5c). Meanwhile, miR-199b-5p restoration or DDR1 silencing can induce a transition from a mesenchymal-like toward a more cobblestone-like epithelial phenotype (Fig. 5a), as supported by the E-cadherin overexpression and the downregulation of vimentin and fibronectin expression in PCa cells and xenograft tumours (Figs. 4a, b, 5b, c). Coherent with the reversion of the mesenchymal phenotype, miR-199b-5p overexpressed cells showed significantly impaired migratory and invasive capabilities compared to the NC cells (Supplementary Fig. S1B–E), indicating the inhibitory effect of miR-199b-5p on EMT traits induced by DDR1 in PCa. Taken together, these results revealed the tumour-suppressive role of miR-199b-5p in PCa context through acting mainly on ERK downstream pathways controlling cell growth, EMT and metastasis. Also, these findings suggested the involvement of miR-199b-5p loss in the initial phases of the metastatic process, specifically EMT, migration and invasion.

### DDR1 expression is upregulated in PCa tissues and correlated with poor clinical outcomes of PCa patients

The clinical and pathologic characteristics of the enrolled PCa patients are shown in Table 1. IHC results revealed that DDR1 expression was present in 28 of 56 (50%) patients with primarily localised PCa and in all 38 (100%) cases with metastatic PCa (*P* < 0.001, Supplementary Fig. S4A). As shown in Table 1, DDR1 expression was negatively correlated with miR-199b-5p expression in clinical specimens. Moreover, DDR1 positive expression was significantly correlated with the high Gleason score (*P* < 0.001), advanced pathological tumour stage (*P* = 0.024) and distal metastases (*P* < 0.001), suggesting a positive association between DDR1 expression and tumour malignant behaviour. Importantly, Kaplan–Meier analysis revealed that PCa patients with positive DDR1 expression had shorter overall survival than those with DDR1 negative expression (*P* = 0.028, Supplementary Fig. S4B). Furthermore, the results from analysing the GEO database (accession number: GSE16560) and the TCGA-PRAD database revealed that PCa patients with elevated DDR1 expression had the shorter overall survival than those with low DDR1 expression (*P* = 0.013 and *P* = 0.036, respectively, Supplementary Fig. S4C). Taken together, these findings demonstrated a promoting action of DDR1 in the clinical progression of PCa.

## Discussion

The growing evidence illustrated that aberrant miRNAs expression have been causatively associated with the invasion and metastasis in PCa. Specifically, the downregulation of a miRNA may result in deregulated overexpression of its targeted oncogene, thus contributing to tumour development and progression.^4^ In this study, we demonstrated that miR-199b-5p levels were significantly reduced in the metastatic PCa tissues and cells when compared with the localised disease, the weakly metastatic and androgen-dependent PCa cell and normal prostate epithelial cell. The results indicated the involvement of miR-199b-5p in regulating the transition from organ-confined PCa to metastatic disease. ADT is a first line of therapy for castration-resistant and metastatic PCa. In this study, loss-of-function and gain-of-function experiments showed that miR-199b-5p had no effects on AR expression in AR-positive and AR-negative PCa cells, suggesting that AR was not involved in miR-199-5p pathway. Moreover, we found that miR-199b-5p overexpression could drastically suppress PCa cell proliferation, migration and invasion in vitro and tumour growth and metastasis in vivo. Up to now, the clinical significance of miR-199b-5p in PCa is still unclear. In this study, by analysing Taylor dataset, we found that PCa patients with low miR-199b-5p expression level had a remarkably shorter overall survival than those with high miR-199b-5p level (*P* = 0.019), suggesting that downregulation of miR-199b-5p could predict the poor prognosis of patients with PCa. Similarly, Fang et al.^23^ reported an obvious association of reduced miR-199b-5p expression with poor prognosis in patients with breast cancer. All these findings revealed that loss of miR-199b-5p contributed greatly to the invasion and metastasis of PCa. Mechanistically, our results showed that miR-199b-5p could downregulate DDR1 expression by directly targeting its 3’-UTR to hinder cell migration, invasion and metastasis, the prelude of which is EMT. Similar results were reported in breast cancer by Wu et al.,^24^ where miR-199b-5p inhibited the proliferative, migratory and invasive capabilities of triple negative breast cancer cells via directly targeting and downregulating DDR1 expression. The study of Zhou et al.^11^ showed that miR-199b-5p/N-Cadherin axis depressed cell migration, invasion and TGF-b1-mediated EMT in hepatocellular carcinoma. EMT is a crucial step for the metastatic dissemination, which represents the main cause of death in PCa patients. Hence, preventing EMT as a form of cellular plasticity is a powerful approach to reduce cancer formation and metastatic spread.^25^ Furthermore, in the study, we observed the supplementation of miR-199b-5p could significantly repress EMT-associated traits of PCa cells both in vitro and in vivo. Interestingly, such phenotype was recapitulated by the direct silencing of DDR1 by shRNA. Altogether, for the first time, we provide evidence of a tumour-suppressive function of miR-199b-5p in PCa invasion and metastasis.

DDR1 is a class of collagen receptor, the upregulation of which has been widely associated with more aggressive behaviour in several malignant tumours including lung cancer, hepatocellular carcinoma, glioma and breast cancer.^24,26–29^ In the context of PCa; however, the clinical significance and the role of aberrant DDR1 expression remain unclear. In the study by Shimada et al.,^30^ it is demonstrated that prostate cancer antigen (PCA)*-*1/DDR1 signalling axis promoted cancer invasion and androgen-independent progression in PCa through upregulating DDR1 expression. Recently, Azizi et al.^19^ reported that a selective DDR1 inhibitor DDR1-IN-1 could reduce cell migration and EMT and induce cell-cycle arrest and apoptosis in PCa DU145 and LNcap-FGC cells. In our study, DDR1 expression was detected in 66 of 94 (70.2%) patients with PCa and was significantly correlated with high Gleason score (*P* < 0.001), advanced pathological tumour stage (*P* = 0.024), distal metastases (*P* < 0.001) and shorter overall survival (*P* = 0.028). Moreover, the results from analysing the public GEO database and TCGA-PRAD database revealed that PCa patients with relative high DDR1 expression had shorter overall survival than those with relative low DDR1 expression (*P* < 0.05 for all). Taken together, these results indicated that DDR1 overexpression contributed greatly to the aggressiveness of PCa. In this study, we also investigated whether DDR1 affects the downstream pathways, and found that DDR1 shRNA or miR-199b-5p could significantly suppress ERK signalling in PCa cells and xenograft tumours. Activation of p-ERK pathway has been reported to induce the invasion, metastasis and EMT in PCa.^20–22,31^ Thus, the findings presented here suggested that miR-199b-5p-DDR1-ERK signalling axis resulted in inhibition of EMT, which ultimately led to suppressed cell growth, invasion and metastasis in PCa. The regulatory roles of DDR1 activating ERK signalling pathway need further investigation to unveil the mechanisms underlying DDR1-mediated EMT in PCa.

In metastatic PCa, a recent study by Wa et al.^32^ reported that miR-19a-3p expression is downregulated in bone metastatic PCa cells and tissues, and that upregulation of miR-19a-3p reduced invasion, migration and bone metastasis formation in PCa by targeting SMAD2/SMAD4/ TGF-β signalling pathway. Bonci et al.^33^ found that loss of miR-15 and miR-16 in combination with an increased expression of miR-21 promoted PCa cells spreading, bone marrow colonisation and bone destruction. In addition, the study by Huang et al.^34^ showed that the level of miR-141-3p expression was lower in bone metastatic PCa tissues than that in non-bone metastatic PCa tissues, and that miR-141-3p overexpression suppressed the invasion, migration and EMT of PCa cells while loss of miR-141-3p promoted the metastatic features of cancer cells. Metastasis is the disseminated growth of tumours in distant organs. As shown in Fig. 2, miR-199b-5p overexpression slowed tumour growth, while downregulating miR-199b-5p promoted tumour growth in both the subcutaneous model and lung metastasis model. These results suggested that tumour growth inhibition by miR-199b-5p might lead to the decreased lung metastasis or the reduced metastatic tumour growth in lung. Taken together with the findings presented here, all these results underscore the notion that miRNAs might be of therapeutic potential in the context of PCa metastases. For the translational exploitation of miR-199b-5p in PCa, a potential clinical interest may also lie on its ability to suppress cell growth and EMT. In this regard, a miR-199b-5p-based therapy in an organ-confined PCa context may primarily represent a suitable strategy to reduce tumour growth and prevent metastases. In this study, we observed in vitro that miR-199b-5p-transfected PCa cells could maintain significantly higher expression levels of miR-199b-5p than NC cells for at least 21 days while anti-miR-199b-5p-transfected cells had significantly reduced miR-199b-5p levels at 7 days after transfection as compared to NC cells. In line with in vitro data, the miR-199b-5p was still about 180-250 times more expressed in the xenografts derived from miR-199b-5p-transfected cells as compared to NC ones whereas xenografts derived from anti-miR-199b-5p-transfected cells maintained significantly lower levels of miR-199b-5p than NC ones, at 28 days after injection into mice. Furthermore, the functional experiments demonstrated the markedly inhibited effects of miR-199b-5p overexpression on the targeted gene and tumour growth, invasion and metastasis. Our results also verified the stability and functionality of the miR-199b-5p and anti-miR-199b-5p-transfected cells in vitro and in vivo. Therefore, the development of any miR-199b-5p-based therapeutic approach as a novel strategy to withstand the disease, alone or in combination with the currently used therapies would be promising in the near future.

In conclusion, the findings presented in this study demonstrated that miR-199b-5p was significantly downregulated in metastatic PCa and exerts tumour-suppressive functions via directly targeting DDR1. Moreover, loss of miR-199b-5p promoted the growth, invasion and metastasis of PCa cells both in vitro and in vivo, which may be attributed to DDR1-induced EMT through activating ERK signalling pathway. Our study identified the miR-199b-5p-DDR1-ERK signalling axis in PCa, which represents a novel mechanism of regulating EMT and supports the potential utility of miR-199b-5p as a promising therapeutic strategy for PCa metastases.

## Supplementary information

Supplementary Materials

## Data Availability

Data pertaining to this study are included in the manuscript. TargetScan, miRDB, miRWalk, miRTarBase, The Cancer Genome Atlas database (TCGA), Gene Expression Profiling Interactive Analysis (GEPIA), and the Gene Expression Omnibus (GEO) datasets used and/or analysed in this work are publicly available from http://www.targetscan.org/, http://mirdb.org/, http://zmf.umm.uni-heidelberg.de/, http://mirtarbase.mbc.nctu.edu/, https://tcgadata.nci.nih.gov/tcga/, http://gepia.cancer-pku.cn/ and http://www.ncbi.nlm.nih.gov/geo/, respectively. Other data that support the findings of this study are available from the corresponding author upon request.
