# Correction: miR-199b-5p-DDR1-ERK signalling axis suppresses prostate cancer metastasis via inhibiting epithelial-mesenchymal transition

**DOI:** 10.1038/s41416-020-01258-w

**Published:** 2021-01-13

**Authors:** Zhigang Zhao, Shankun Zhao, Lianmin Luo, Qian Xiang, Zhiguo Zhu, Jiamin Wang, Yangzhou Liu, Jintai Luo

**Affiliations:** 1 Department of Urology and AndrologyThe First Affiliated Hospital of Guangzhou Medical University;, Guangdong Provincial Key Laboratory of Urology, 510230 Guangzhou, China; 2 Taizhou Central Hospital, Taizhou, China

**Keywords:** Oncogenes, Prostate cancer

**Correction to:** *British Journal of Cancer* (2020); 10.1038/s41416-020-01187-8, published online 26 November 2020

Since the publication of this paper, the authors have noticed an error in the name of the Institution for Dr. Shankun Zhao. His Institution should be Taizhou Central Hospital (Taizhou University Hospital) and not Zhejiang Taizhou Central Hospital (Affiliated Hospital of Taizhou University). This has been corrected above. The authors apologise for this oversight.

