# Supplementary material for: miR-199b-5p-DDR1-ERK signalling axis suppresses prostate cancer metastasis via inhibiting epithelial-mesenchymal transition
Source: Br J Cancer. 2020 Nov 26;124(5):982–94. doi: 10.1038/s41416-020-01187-8 (PMC7921430; doi:10.1038/s41416-020-01187-8)
Supplement: Supplementary file 1 — Supplementary Materials [file 41416_2020_1187_MOESM1_ESM.docx]

**Supplementary Fig. S1**

**
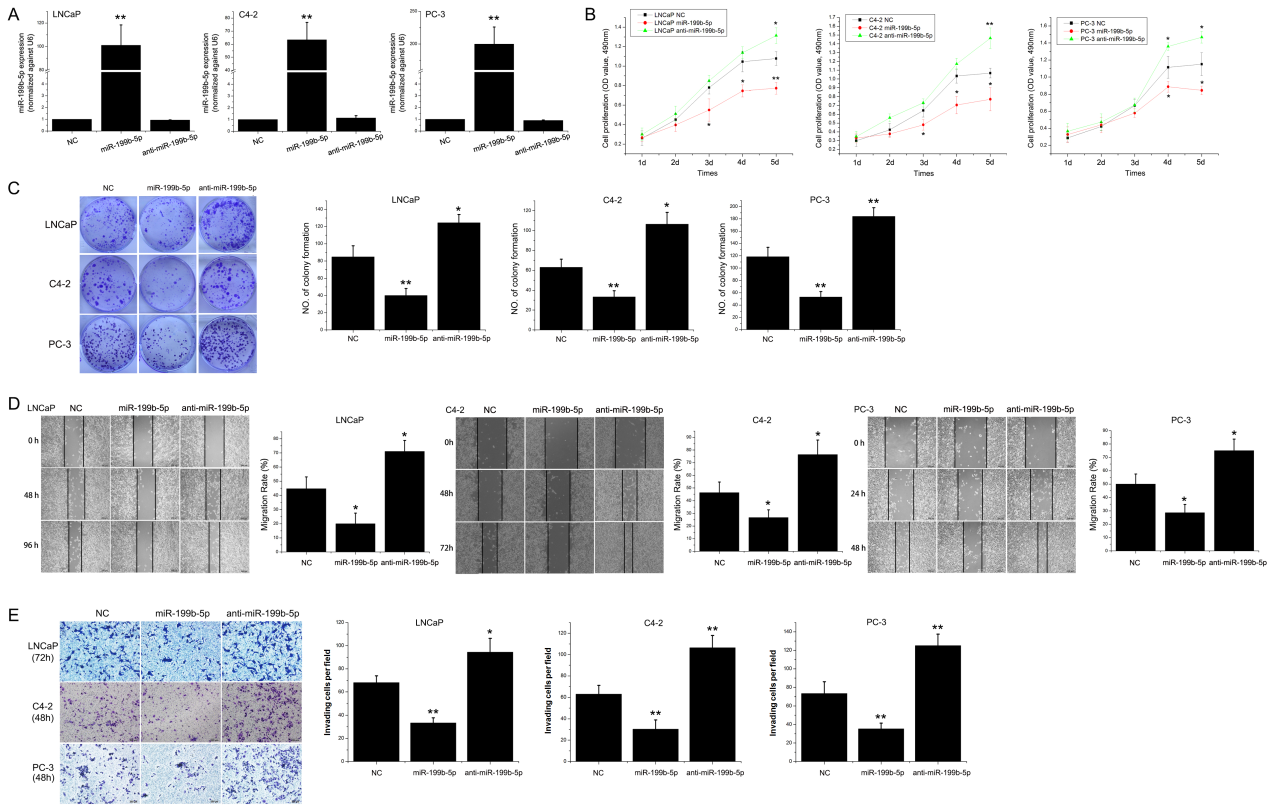
**

**Supplementary Fig. S2**

**
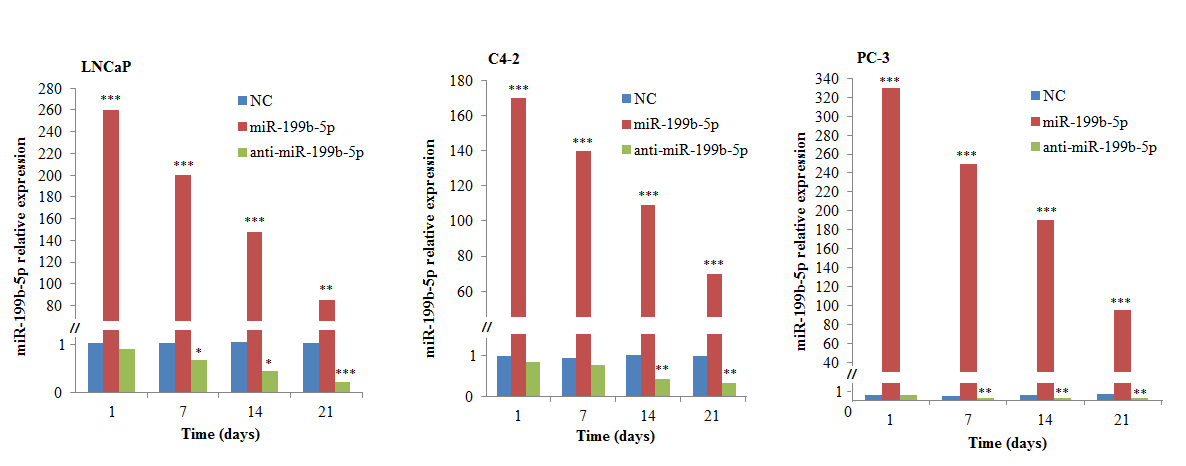
**

**Supplementary Fig. S3**

**
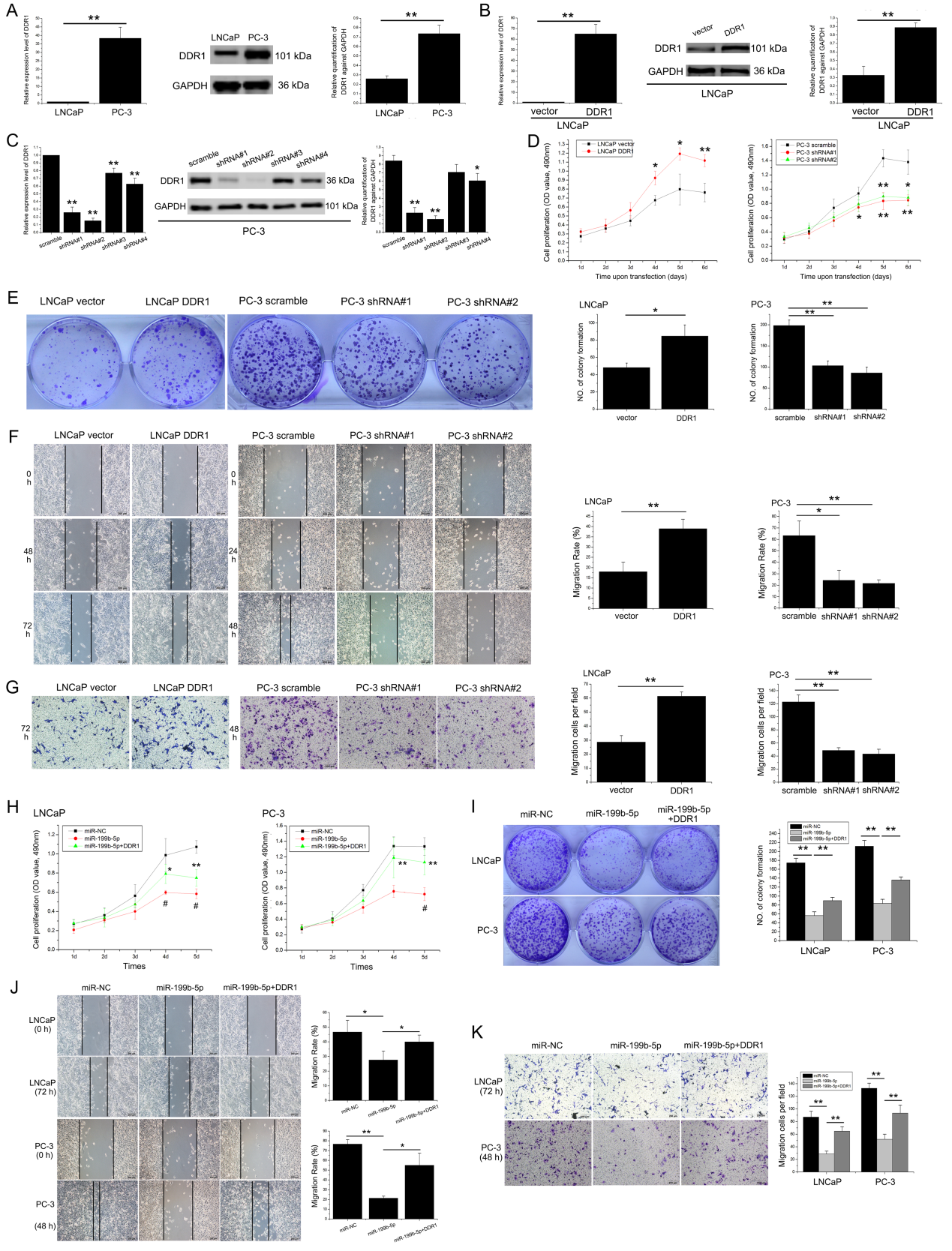
**

**Supplementary Fig. S4**

**
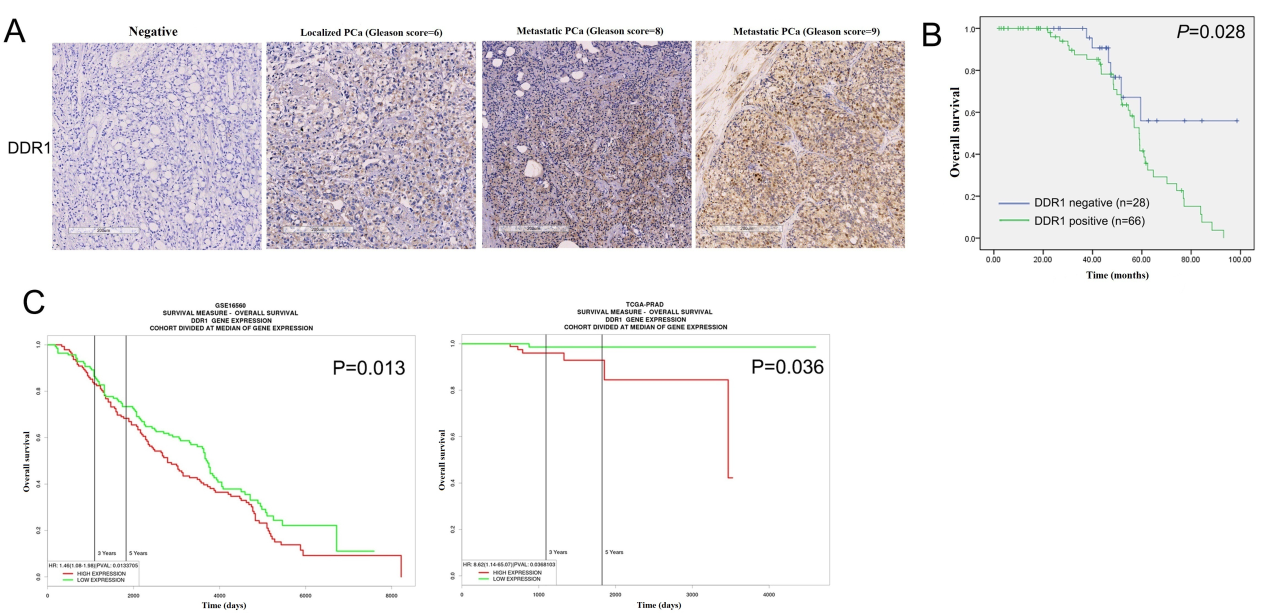
**

**Supplementary Figure Legends**

**Supplementary Fig. S1** **miR-199b-5p reduces proliferation, migration and invasion of PCa cells in vitro.**

1. Relative expression levels of miR-199b-5p were detected by qRT-PCR assay in PCa cells stably transfected with miR-199b-5p, anti-miR-199b-5p, and NC at 3 days after transfection. **B.** Graphs showed the growth of miR-199b-5p, anti-miR-199b-5p, and NC transfected PCa cells at difffferent time points using MTT assay. **C.** The number of colony formation of transfected cells was evaluated using colony formation assay. **D.** The migration ability of transfected cells was assessed by wound-healing assay measuring gap distance at difffferent time points. **E.** The number of transfected cells across the membrane and the invasion capability were evaluated using transwell assays. The results are reported as the mean ± SD of three independent experiments. *P < 0.05 and **P < 0.01.

**Supplementary Fig. S2 The stability of miR-199b-5p and anti-miR199b-5b transfected PCa cells is evaluated in vitro.**

qRT-PCR analysis showed the relative expression of miR-199b-5p in miR-199b-5p and anti-miR199b-5b transfected LNCaP, C4-2 and PC-3 cells with respect to NC cells, at different time points after transfection, respectively. U6 was used as endogenous control. *P < 0.05, **P < 0.01 and ***P < 0.001.

**Supplementary Fig. S3 DDR1 reverses the inhibitory effects of miR-199b‑5p in PCa cells.**

**A.** qRT-PCR assay showed that the expression level of DDR1 mRNA was remarkably higher in PC-3 cell than that in LNCaP cell (left). Western blotting assay (middle) and its quantification analysis by grey scale (right) confirmed a higher expression level of DDR1 protein in PC-3 cell than in LNCaP cell. **B.** qRT-PCR assay (left), western blotting assay (middle) and grey scale analysis (right) showed DDR1 over-expression in LNCaP cells with transfecting pcDNA3.1-DDR1. **C.** qRT-PCR assay (left), western blotting assay (middle) and grey scale analysis (right) showed DDR1 down-regulation in PC-3 cells with transfecting LV16-shRNA-DDR1 (shRNA#1 or shRNA#2). **D.** Graphs showed the growth of LNCaP cells with DDR1 over-expression (left) and PC-3 cells with DDR1 silence (right) at different time points using MTT assay. **E.** Colony formation assay was performed to evaluate the effects of DDR1 expression on the number of colony formation in LNCaP (left) and PC-3 cells (right). **F.** Wound-healing assay was used to assess the effects of DDR1 expression on migration ability of LNCaP (left) and PC-3 cells (right) by measuring gap distance at different time points. **G.** Transwell assays were carried out to evaluate the effects of DDR1 expression on the number of LNCaP (left) and PC-3 (right) cells across the membrane and the invasion capability. **H-K.** MTT (H), colony formation (I), wound-healing (J) and transwell (K) assays showed that the restored expression of DDR1 reversed the suppresive effect of miR-199b-5p on the growth, colony formation number, migration and invasion capabilities of LNCaP and PC-3 cells. The results are reported as the mean ± SD of three independent experiments. *P < 0.05 and **P < 0.01.

**Supplementary Fig. S4 DDR1 is over-expressed in PCa tissues and correlated with poor clinical outcomes of PCa patients.**

**A.** Representative IHC staining showed the expression status of DDR1 in clinically localized and metastatic PCa tissues. Scale bar, 200 µm. **B.** Kaplan-Meier analysis showed a significantly shorter overall survival in PCa patients with positive DDR1 expression than those with negative DDR1 expression. **C.** Bioinformatics analyses from the GEO database (accession number: GSE16560) (left) and the TCGA-PRAD database (right) showed the shorter overall survival in PCa patients with high DDR1 expression than those with low DDR1 expression, respectively.

Supplementary Table S1. Description of the primer sequences used in this study

|  | Sequences |  |
| --- | --- | --- |
|  | Forward | Reverse |
| hsa-miR-199b-5p | 5’TGCCCAGTGTTAGACTATCTGTTCTTCAAGAGAGAACGATTCTAAACACTGGGTTTTTTC-3’ | 3’ACGGGTCACAAATCTGATAGACAAGAAGTTCTCTCTTGTCEAAGATTTGTGACCCAAAAAAGAGCT-5’ |
| hsa-anti-miR-199b-5p | 5’TGAACAGATAGTCTAAACACTGGGTTCAAGAGACCCAGTCTAGACTATCTGTTCTTTTTTC-3’ | 3’ACTTGTCTATCAGATTTGTGACCCAAGTTCTCTGGGTCACATCTGATAGACAAGAAAAAACAGCT-5’ |
| hsa-miR-NC | 5’TGTTCTCCGAACGTGTCACGTTTCAAGAGAACGTGACACGTTCGGAGAACTTTTTTC-3’ | 3’ACAAGAGGCTTGCACAGTCAAAGTTCTCTTGCACTGTGCAAGCCTCTTGAAAAAAG-5’ |
| CCNL1 | TACCATCGACCACTCTCTGATT | GGATGCGTAAGTCCGTCTCAC |
| DDR1 | AAGGGACATTTTGATCCTGCC | CCTTGGGAAACACCGACCC |
| GAPDH | CGCTGAGTACGTCGTGGAGTC | GCTGATGATCTTGAGGCTGTTGTC |
